# The Approach for Action Recognition Based on the Reconstructed Phase Spaces

**DOI:** 10.1155/2014/495071

**Published:** 2014-11-10

**Authors:** Hong-bin Tu, Li-min Xia

**Affiliations:** School of Information Science and Engineering, Central South University, Hunan 410075, China

## Abstract

This paper presents a novel method of human action recognition, which is based on the reconstructed phase space. Firstly, the human body is divided into 15 key points, whose trajectory represents the human body behavior, and the modified particle filter is used to track these key points for self-occlusion. Secondly, we reconstruct the phase spaces for extracting more useful information from human action trajectories. Finally, we apply the semisupervised probability model and Bayes classified method for classification. Experiments are performed on the Weizmann, KTH, UCF sports, and our action dataset to test and evaluate the proposed method. The compare experiment results showed that the proposed method can achieve was more effective than compare methods.

## 1. Introduction

Automatic recognition of human actions from image sequences is a challenging problem that has attracted the attention of researchers in the past decades. This has been motivated by the desire for application of entertainment, virtual reality, motion capture, sport training [1–3], medical biomechanical analysis, and so on.

In a simple case, where a video is segmented to contain only one execution of a human activity, the objective of the system is to correctly classify the video into its activity category. More generally, the continuous recognition of human activities must be performed by detecting the starting and ending times of all occurring activities from an input video. Aggarwal and Ryoo [4] summarized the general method as single-layered approaches, hierarchical approaches, and so forth. Single-layered approaches represent and recognize human activities directly based on sequences of images. So, they are suitable for the recognition of gestures and actions with sequential characteristics. Single-layered approaches are again classified into two types: space-time approaches and sequential approaches. Space-time approaches are further divided into three categories: space-time volume, trajectories, and space-time features. Hierarchical approaches represent high-level human activities by describing them in terms of simpler activities. Hierarchical approaches usually can be divided into 3 classes: the statistical, the syntactic, and the description-based classes. Recognition systems composed of multiple layers are constructed, which are suitable for the analysis of complex activities. Among all these methods, the space-time approaches are the most widely used ones to recognize simple periodic actions such as “walking” and “waving,” and periodic actions will generate feature patterns repeatedly and the local features are scale, rotation, and translation-invariant in most cases. However, the space-time volume approach is difficult in recognizing the actions when multiple persons are present in the scene and it requires a large amount of computations for the accurate localization of actions. Besides, it is difficult in recognizing actions which cannot be spatially segmented. The major disadvantage of the space-time feature is that it is not suitable for modeling more complex activities. In contrast, the trajectory-based approaches have the ability to analyze detailed levels of human movements. Furthermore, most of these methods are view-invariant. Therefore, the trajectory-based approaches have been the most extensively studied approaches.

Several approaches used the trajectories themselves to represent and recognize actions directly. Sheikh et al. [5] applied a set of 13 joint trajectories in a 4D XYZT space to describe the human action. Yilmaz and Shah [6] presented a methodology to compare action videos by the set of 4D XYZT joint trajectories. Anjum and Cavallaro [7] proposed algorithm based on the extraction of a set of representative trajectory features. Jung et al. [8] designed the novel method to detect event by trajectory clustering of objects and 4D histograms. Hervieu et al. [9] used Hidden Markov models to capture the temporal causality of object trajectories for the unexpected event detection. Wang et al. [10] proposed a nonparametric Bayesian model to analysis trajectory and model semantic region in surveillance. Wang et al. [11] presented a video representation based on dense trajectories and motion boundary descriptors for recognizing human actions. Yu et al. [12] used the novel approach based on weighted feature trajectories and concatenated bag-of-features (BOF) to recognize action. Pao et al. [13] proposed a general user verification approach based on user trajectories, which include on-line game traces, mouse traces, and handwritten characters. Yi and Lin [14] introduced the salient trajectories to recognize. Du et al. [15] proposed an intuitive approach on videos based on the feature trajectories. Psarrou et al. [16] designed the model of the statistical dynamic to recognize human actions by learning prior and continuous propagation of trajectories models.

These approaches approximated the true motion state by setting constraints on the type of the dynamical model [1]. Above all, they required the detailed mathematical and statistical modeling. To solve these problems, we present the approach for action recognition based on the reconstructed phase spaces.

The remainder of this paper is organized as follows.  Section 2 presents the modified particle filter that is used to track human key points. In Section 3, we reconstruct the phase space of the total data.  Section 4 explains the probability generation model.  Section 5 explains action classification.  Section 6 explains the results and analysis of the proposed approach. Finally, we conclude the paper in Section 7.

## 2. Human Key Joints Track

**Figure 1:**
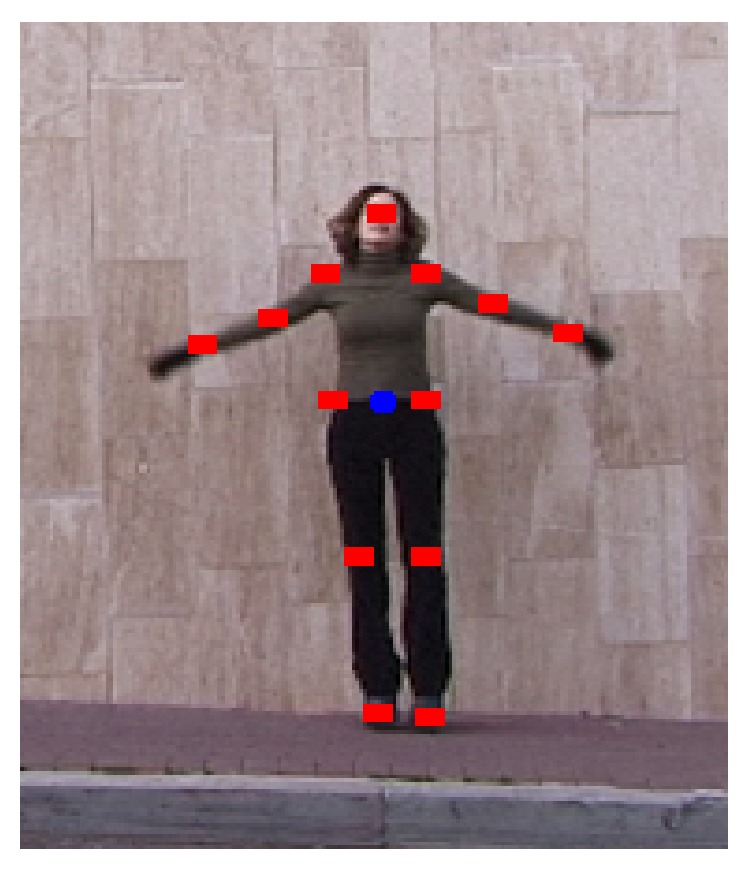
The human joints model. The original photo stems from Weizmann dataset [21].

The human body [2] is divided into 15 key points, which are named 15 key joint points for representing the human body structure (torso, pelvis, left upper leg, left lower leg, left foot, right upper leg, right lower leg, right foot, left upper arm, left lower arm, left hand, right upper arm, right lower arm, right hand, and head) [17], which the 15 joints trajectory represents the human body behavior (blue dot represents pelvis, which is the origin of coordinate). Another consideration was that these joints were relatively easy to automatically detect and track in real videos, as opposed to the inner body joints which were more difficult to track. Each key joint had a trajectory as the time was going on and 15 trajectories were used to represent different actions. Therefore, we must track accurately the human body 15 nodes for indicating the human behavior. These are illustrated in Figure 1.

However, it is difficult to track some key points for occlusion. In this paper, we use the modified particle filters to track these key points. Particle filters are very efficient methods for tracking multiple objects, which they can cope with no-linear and multimodality induced by occlusions and background clutter. But it has been proved that the number of samples increases exponentially with the size of the state vector to be explored. The reason is that one sample dominates the weight distribution and the rest of the samples are not in statistically significant regions. In order to solve the above problem, we adopt the integrated algorithm based on both particle filters and Markov chain Monte Carlo models [18, 19], which is based on drift homotopy for stochastic differential equations and the existing particle filter methodology for multitarget tracking by appending an MCMC step after the particle filter resampling step. The MCMC step is integrated to the particle filter algorithm to bring the samples closer to the observation at the same time respecting the target dynamics.

We can assume [18] the parameters as follows: *Z*_*K*_1__,…, *Z*_*K*_*N*__: the noisy observations, *K*_1_,…, *K*_*N*_: the status of the system particular time, *Z*_*K*_*n*__ = *G*(*X*_*K*_*n*__, *η*_*n*_) (*η*_*n*_, *n* = 1,…, *N*): the observations functions, *g*(*X*_*K*_*n*__, *Z*_*K*_*n*__): the distribution of the observations, and *E*[*f*(*X*_*K*_*n*__∣{*Z*_*K*_*n*__}_*i*=1_^*N*^]: the conditional expectation.

Given a video sequence and labeled samples of object or background pixels on the first frame [20], we have access to noisy observations of the status of the system particular time.

The filtering problem consists of computing estimates of the conditional expectation. Therefore, we can compute the conditional density of the state of the system *p*(*X*_*T*_*k*__∣{*Z*_*Z*_*k*__}_*i*=1_^*k*^) and define a reference density: *q*(*X*_*K*_*n*__∣{*Z*_*K*_*n*__}_*n*=1_^*N*^). At last, we obtain the weighted sample [18]:

(1) EfXKn ∣ ZKni=1N  ∝1N∑n=1NfXKnpXKn ∣ ZKni=1N∑n=1NqXKn ∣ ZKni=1N,

(2) $$\begin{array}{c} p\left(X_{K_{n}}\;|\;{\left\{Z_{K_{n}}\right\}}_{n=1}^{N}\right)\propto \frac{g\left(X_{K_{n}},Z_{K_{n}}\right)p\left(X_{K_{n}}\;|\;{\left\{Z_{K_{i}}\right\}}_{i=1}^{N}\right)}{q\left(X_{K_{n}}\;|\;{\left\{Z_{K_{i}}\right\}}_{i=1}^{N}\right)}, \end{array}$$

(3) pXKn ∣ ZKii=1N  =∫pXKn ∣ XKn−1pXKn−1 ∣ ZKii=1N−1dXKi−1.

We assume that

(4) $$\begin{array}{c} q\left(X_{K_{n}}\;|\;{\left\{Z_{K_{i}}\right\}}_{i=1}^{N}\right)\propto p\left(X_{K_{n}}\;|\;{\left\{Z_{K_{i}}\right\}}_{i=1}^{N-1}\right), \end{array}$$

and, from (2), we can obtain the formula

(5) $$\begin{array}{c} \frac{p\left(X_{T_{k}}\;|\;{\left\{Z_{T_{i}}\right\}}_{i=1}^{k-1}\right)}{q\left(X_{T_{k}}\;|\;{\left\{Z_{T_{i}}\right\}}_{i=1}^{k}\right)}\propto g\left(X_{K_{n}},Z_{K_{i}}\right). \end{array}$$

The approximation in expression (1) becomes

(6) EfXKn ∣ ZKnn=1N  ≈∑n=1NfXKnngXKnn,ZKn∑n=1NqXKn ∣ ZKnn=1NgXKnn,ZKn.

Thus, we can define the (normalized) weights

(7) $$\begin{array}{c} w_{K_{n}}^{n}=\frac{q\left(X_{K_{n}}\;|\;{\left\{Z_{K_{i}}\right\}}_{i=1}^{N}\right)g\left(X_{K_{n}}^{n},Z_{K_{i}}\right)}{\sum_{n=1}^{N}g\left(X_{K_{n}}^{n},Z_{K_{i}}\right)}. \end{array}$$

The tracking algorithm is described as follows.

(1) Sampling *N* particles in accordance with the unified weights randomly generated particles form unweighted samples *X*_*K*_*n*−1__^*n*^ and determination *p*(*X*_*K*_*n*__∣{*Z*_*K*_*n*−1__}_*n*=1_^*N*−1^), as follows:
  (8) $$\begin{array}{c} p\left(X_{K_{n}}\;|\;{\left\{Z_{K_{n-1}}\right\}}_{n=1}^{N-1}\right)=\overset{\wedge }{\underset{\lambda =1}{\prod }}p\left(X_{K_{\lambda },K_{n}}\;|\;{\left\{Z_{\lambda ,K_{n}-1}\right\}}_{n=1}^{N-1}\right). \end{array}$$
(2) Predict by sampling *X*_*K*_*n*__^*N*^ from
  (9) $$\begin{array}{c} p\left(X_{K_{n}}\;|\;X_{K_{n-1}}\right)=\overset{\wedge }{\underset{\lambda =1}{\prod }}p\left(X_{\lambda ,K_{n}}\;|\;X_{\lambda ,K_{n-1}}\right). \end{array}$$
(3) Target observation association.
(4) Update and evaluate the weights:
  (10) $$\begin{array}{c} w_{T_{k}}^{n}=\frac{\prod_{\lambda =1}^{\wedge }q\left(X_{K_{n}}\;|\;{\left\{Z_{K_{i}}\right\}}_{i=1}^{N}\right)g_{\lambda }\left(X_{\lambda ,T_{k}}^{m},Z_{\lambda ,T_{i}}\right)}{\sum_{n=1}^{N}\prod_{\lambda =1}^{\wedge }g_{\lambda }\left(X_{\lambda ,T_{k}}^{m},Z_{\lambda ,T_{i}}\right)}. \end{array}$$
(5) By resampling, through the above steps, we can generate independent uniform random variables {*θ* ^*i*^}_*i*=1_^*N*^ (0 < {*θ* ^*i*^}_*i*=1_^*N*^ < 1). Therefore, we can obtain the following equation:
  (11) $$\begin{array}{c} \left(X_{K_{n-1}}^{n},X_{K_{n}}^{n}\right)=\left(X_{K_{n-1}}^{\prime j},X_{K_{n}}^{\prime j}\right), \end{array}$$
  where ∑_*n*=1_^*j*−1^ *w*_*T*_*k*__^*n*^ ≤ *θ* ^*j*^ ≤ ∑_*n*=1_^*j*^ *w*_*T*_*k*__^*n*^.
(6) By Markov chain Monte Carlo tracking, we choose a modified drift for *n* = 1,…, *N* and *k* = 1,…, *K*. Construct a Markov chain [18–20] for *Y*_*K*_*n*__^*N*^ with initial value *X*_*K*_*n*__^*N*^ (the global state of the system is defined by *X*_*K*_*n*__^*N*^) and obtain the stationary distribution
  (12) $$\begin{array}{c} \overset{\wedge }{\underset{\lambda =1}{\prod }}g_{\lambda }\left(Y_{\lambda }^{n},Z_{K_{i}}\right)p_{\lambda }\left(Y_{\lambda }^{n}\;|\;X_{{}_{K_{n}}}^{n}\right). \end{array}$$
(7) Set *X*_*K*_*n*__^*N*^ = *Y*_*K*_*n*__^*N*^.
(8) Set *n* = *n* + 1 and go to Step 1.

**Figure 2:**
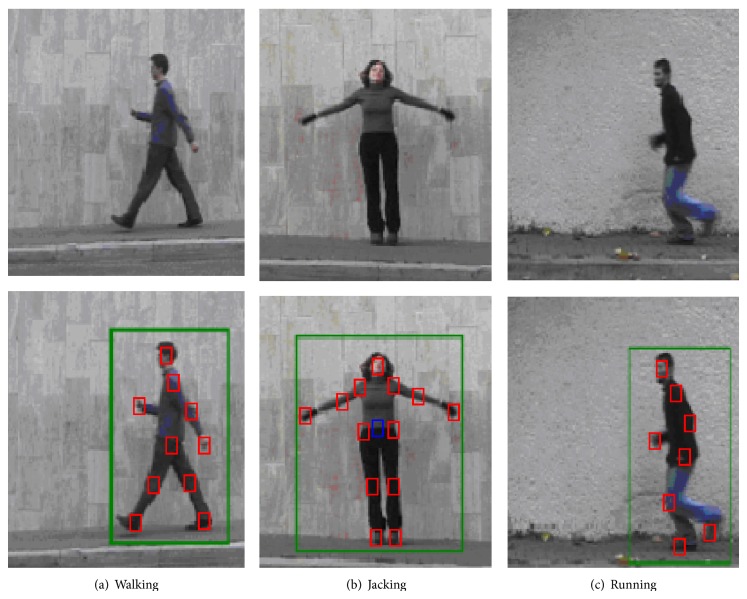
The target tracking results. The original photo stems from Weizmann datasets [21].

Using the tracking algorithm, we can obtain key points trajectories, which are used to recognize human behavior.  Figure 2 depicts the results of human target tracking.

## 3. Phase Space Reconstruction

At present, the phase space reconstruction has been used in many research fields. de Martino et al. [22] constructed the trajectory space and refer to the phase space in the dynamic system. Paladin and Vulpiani [23] presented the embedding trajectory dimension, which was similar to reconstruct the embedding dimension of the phase spaces of the dynamic system. Fang and Chan [24, 25] present the unsupervised ECG-based identification method based on phase space reconstruction in order to save the picking up characteristic points. Nejadgholi et al. [26] used the phase space reconstruction for recognizing the heart beat types. In this paper, we use the phase space reconstruction for human action recognition.

We use the linear dynamic systems instead of the traditional gradient and optical flow features of interest points to recognize action. The linear dynamic system [27] is suitable to deal with temporally ordered data, which has been used in several applications in computer vision, such as tracking, human recognition from gait, and dynamic texture. The temporal evolution of a measurement vector can be modeled by the dynamic system. In this case, we use the linear dynamic system to model the spatiotemporal model. In this series, it is sometimes necessary to search for patterns not only in the time series itself, but also in a higher-dimensional transformation of the time series. We can estimate the delay time *τ* and embedding dimensions *d* in reconstructed phase space in order to extract more useful information from human action trajectories. These parameters can be computed as follows.

The phase portrait of a dynamic system [28] described by a one-dimensional time series of measured scalar values *x*(*t*) can be reconstructed in a *k*-dimensional state space. From the time-series signal, we can construct an *m*-dimensional signal *x*(*t*). We define [28] a dynamical system as the possibly nonlinear map, which represents the temporal evolution of state variables

(13) $$\begin{array}{c} x\left(t\right)=\left[x_{1}\left(t\right),x_{1}\left(t\right),\ldots ,x_{m}\left(t\right)\right]\in R^{m}. \end{array}$$

de Martino et al. [22] pointed out that the phase space reconstruction based on Taken's theory is equivalent to the original attractor if *m* is large enough by suitable hypotheses.

Each point in the phase space is calculated according to [26]. Consider

(14) $$\begin{array}{c} x_{n}=\left[x_{n}x_{n-\tau },\ldots ,x_{n-(d-1)\tau }\right]n=\left(1+\left(d-1\right)\tau \right),\ldots ,N, \end{array}$$

where *x*_*n*_ is the *n*th point in the time series, delay times *τ* is the time lag, *N* is the number of points in the time series, and *d* is the dimension of the phase space. From now on, *η* is used to denote this set of body model variables describing human motion.

The reconstructed phase space is shown by López-Méndez and Casas and Takens [28, 29] for the large enough *m*, which is a homeomorph *m* (embedding dimension) of the true dynamical system in the generated time series. We used Takens' theorem to reconstruct state spaces by time-delay embedding. In our case, parameters [26, 28] are defined as follows:

 
*η*: the temporal evolution;
 
*Y*_*K*_*n*__^*N*^: time series (scalar), and we want to characterize
  (15) $$\begin{array}{c} Y_{K_{n}}^{{}^{\eta }N}=\left[z^{\eta }\left(t\right),z^{\eta }\left(t+\tau \right),\ldots ,z^{\eta }\left(t+\left(m-1\right)\tau \right)\right]. \end{array}$$

*Y*_*K*_*n*__^^*η*^*N*^ is a point in the reconstructed phase space, *m* is the embedding dimension, and *τ* is the embedding delay. Therefore, the phase space can be reconstructed by stacking sets of *m* (the large enough *m*) temporally spaced samples. The embedding delay *τ* determines the properties of the reconstructed phase space.

At first, the embedding delay using the mutual information method was determined [26] and the estimated delay was used to obtain the appropriate embedding dimension [30]. Once both the embedding delay and the embedding dimension have been estimated, is performed [26] as follows:

(16) x^η=YKnηN1+d−1τ−YKnηN2+d−1τ−⋮YKnηNn+d−1τ−=zη1+d−1τ0−,…,zη1+d−1ττ−,…,zη1+d−1τm−1τ−zη2+d−1ττ−,…,zη2+d−1t+τ−,…,zη2+d−1t+m−1τ−⋮zηNN−1−m−1τ−,…,zηNN−1−m−2τ−,…,zηNN−1−.

We use the phase space ${\hat{x}}^{\eta }$ as signatures, where each one of the model variables constitutes a time series from the reconstructed phase space. The time series [28] model provides a better performance to recognize the action model based on independent scalar time series, which are based on action recognition method. Therefore, we get the phase space corresponding to each point trajectory, which contained the joint point of occlusion and nonocclusion. Besides, we choose Kolmogorov-Sinai entropy [31, 32] as another feature for analyzing the dynamics human action. *K*-S entropy (HKS) is the average entropy per unit time. We define it as the following [32]:

(17) $$\begin{array}{c} H_{k}=\frac{\lim_{\kappa \to 0}\lim_{t\to \infty }\left[\sum_{i=1}^{N_{t}}P_{\kappa }\sum_{}^{}\log\left(1/P_{k}\right)\right]}{t}. \end{array}$$

Therefore, each trajectory of the human action can be described as the 3-dimensional feature vector according to the 9-dimensional feature vector of each key joint and 90-dimensional feature vector of each action.

**Figure 3:**
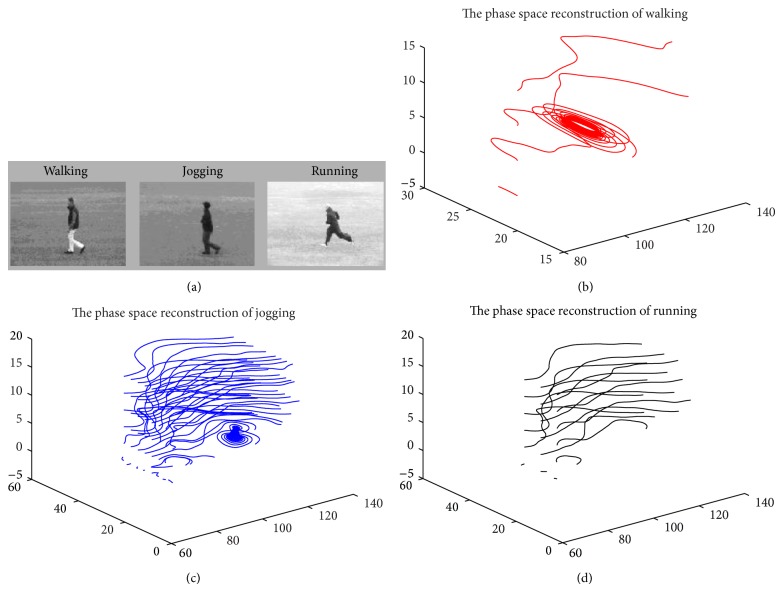
Examples of the reconstructed phase space of the missing data. Hip rotations for walking, jogging, and running actions in the KTH dataset [33]. (a) shows original images. (b) shows the result of reconstructing the reconstructed phase space of the missing data. (b1) shows the phase space reconstruction of right foot motion. (b2) shows the phase space reconstruction of right elbow motion. (b3) shows the phase space reconstruction of right elbow motion. (c) shows the reconstructed phase space of the total occlude joint point. (c1) shows the phase space reconstruction of walking. (c2) shows the phase space reconstruction of jogging. (c3) shows the phase space reconstruction of running.

Figure 3 shows the reconstructed phase space of the total joint point.

## 4. Probability Generation Model

These are a few labeled actions; however, a large number of unlabeled actions need be recognized. Therefore, we use the semisupervised probability model.

It is assumed that [34] the action is generated by a mixture generative model of distribution function *p*(*x*∣*θ*_*i*_). Then, we can obtain the generative model [34] as follows:

(18) $$\begin{array}{c} p\left(x|\theta_{i}\right)=\overset{c}{\underset{i=1}{\sum }}p\left(\theta_{i}\right)p_{i}\left(x|\theta_{i}\right). \end{array}$$

It is generally assumed that the distribution of the feature space is almost consistent with a Gaussian distribution or a multinomial distribution for human action images. *x* is the feature vector of the training sample, *p*(*θ*_*i*_) is the probability of the sample belonging to the *i*th class, *θ*_*i*_ represents the object classes and the covariance matrix of pixel. Therefore, likelihood functions [34] were defined as follows:

(19) log⁡pθi ∣ X=log⁡∏i=1Mpθipxi ∣ θi∏i=M+12Mpxi ∣ θ=∑i=1Mlog⁡pθipxi ∣ θi+∑i=M+12Mlog⁡pXi ∣ θ.

The first part is supervised classification and the second is called unsupervised part.

Unsupervised part should be written as

(20) $$\begin{array}{c} \overset{2M}{\underset{i=l+1}{\sum }}\log p\left(x_{i}\;|\;\theta \right)=\overset{2M}{\underset{i=l+1}{\sum }}\overset{c}{\underset{j=1}{\sum }}p\left(x\right)p\left(x_{i}\;|\;\theta_{i}\right). \end{array}$$

Finally, we can obtain the log-likelihood function

(21) log⁡pθi ∣ X=log⁡∏i=1Mpθipxi ∣ θi∏i=M+12Mpxi ∣ θ +∑i=l+12M∑j=1cpxpxi ∣ θi.  

In this case, we build the relationship between the unlabeled samples and the learning sample. EM is also an iterative algorithm which has two main steps: expectation and maximization.

*E*-step: this step predicts the labels of each unlabeled sample by calculating from the last iteration parameters in (21)

(22) $$\begin{array}{c} p_{ji}^{M}=p\left(\theta_{i}^{M}\;|\;X_{i}\right)=\frac{\theta_{i}^{M}p\left(x_{i}\;|\;\theta_{i}^{M-1}\right)}{\sum_{j=1}^{M}\theta_{i}^{M-1}p\left(x_{i}\right)p\left(x_{i},\theta_{i}^{M-1}\right)}, \end{array}$$

where *p*_*ji*_^*M*^ is the current prediction of model *i* unlabeled samples conditioned on the current distributed parameter,* M*−1 is the previous state value, and *M* is the current state value.

*M*-step: we calculate the current parameters by maximizing the likelihood function as follows:

(23) $$\begin{array}{c} p_{ji}^{M}\left(i,j\right)=\frac{\sum_{k=k+1}^{M}p_{ji}^{M-1}+M_{j}}{M+l},\\ \mu_{j}^{M}=\frac{\sum_{k=1}^{M}p_{ji}^{M-1}x_{k}+\sum_{k=1}^{u_{j}}X_{jk}^{\prime }}{\sum_{k=1}^{M_{j}}P_{jk}+up_{}^{M-1}\left(\theta_{i}\right)+l_{M}},\\ \overset{\left(M\right)}{\underset{j}{\sum }}=\frac{\sum_{k=1}^{2M}p_{jk}^{M-1}\text{CO}{\text{V}}_{j}\left(x_{k}\right)+\sum_{k=1}^{u_{j}}\text{CO}{\text{V}}_{j}\left(x_{jk}^{\prime }\right)}{\sum_{k=1}^{M}P_{jk}+up_{}^{M-1}\left(\theta_{i}\right)+l_{j}}, \end{array}$$

where *p* ^*M*^(*θ*_*i*_) is the posterior distribution of the *k* category, COV_*j*_(•) is the covariance matrix, *u* is the number of unlabeled sample, *j* is the number of the label sample, *l*_*j*_ is the number of the label sample within the *j* class, and *x*_*jk*_′ is the *k* label sample within the *j* class. When the change of the likelihood function between two iterations goes below the threshold, we stop the iteration and export the parameters. Threshold is determined empirically as 0.06.

## 5. Action Classification

We can recognize the human action by trained classified samples by the Bayes classified method [35, 36]:

(24) $$\begin{array}{c} p\left(Y_{i}\;|\;X_{j}\right)=\frac{p\left(X_{j}\;|\;Y_{i}\right)}{\sum_{}p\left(X_{j}\;|\;Y_{i}\right)p\left(Y_{i}\right)}. \end{array}$$

Because our generation model is based on the assumption of a Gaussian mixture distribution, we can obtain the following equation:

(25) pXi ∣ Yj=12π∑p(Yj ∣ Xi) ×exp⁡12X−μYT∑−1Y−μY,

where *μ*_*Y*_ is mean vector and ∑*p*(*Y*_*j*_∣*X*_*i*_) is the covariance matrix. The operation of the classifier is shown in Algorithm 1.

Therefore, we obtain the result of human recognition as follows:

(26) $$\begin{array}{c} Y=\text{ar}{\text{g}}_{Y}\max p\left(Y_{i}|X_{j}\right). \end{array}$$

## 6. Experimental Result

**Figure 4:**
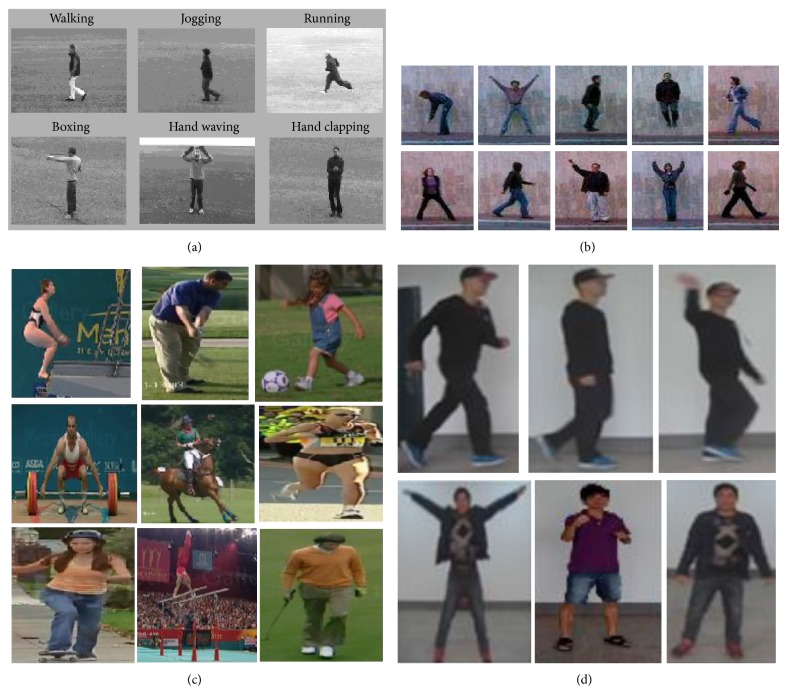
Sample frames from our datasets. The action labels in each dataset are as follows: (a) KTH dataset [33]: walking (a1), jogging (a2), running (a3), boxing (a4), and hand clapping (a5); (b) Weizmann dataset [21]: bending (a1), jumping jack (a2), jumping forward on two legs (a3), jumping in place on two legs (a4), running (a5), galloping sideways (a6), walking (a7), waving one hand (a8), and waving two hands (a9); (c)UCF sports action dataset [37]: diving(a1), golf swinging (a2), kicking (a3), lifting (a4), horseback riding (a5), running (a6), skating (a7), swinging (a8), and walking (a9); (d) our action dataset: walking (a1), jogging (a2), running (a3), boxing (a4), and handclapping (a5).

In this section, firstly, four action datasets are used for evaluating the proposed approach: Weizmann human motion dataset [21], the KTH human action dataset [33], the UCF sports action dataset [37], and our action dataset (Table 8). Secondly, we compare our method with some other popular methods under these action datasets. We use a Pentium 4 machine with 2 GB of RAM, and the implementation on MATLAB to experiment, similar to [3]. Representative frames of this dataset are shown in Figure 4.

### 6.1. Evaluation on KTH Dataset

The KTH dataset is provided by Schuldt which contains 2391 video sequences with 25 actors showing six actions. Each action is performed in 4 different scenarios, which contain some human actions (walking (a1), jogging (a2), running (a3), boxing (a4), and hand waving (a5)).

Representative frames of this dataset are shown in Figure 4(a). The classified results are shown in Table 1.

### 6.2. Evaluation on Weizmann Dataset

The Weizmann dataset is established by Blank, which contains 83 video sequences, showing nine different people, with each performing nine different actions including bending (a1), jumping jack (a2), jumping forward on two legs (a3), jumping in place on two legs (a4), running (a5), galloping sideways (a6), walking (a7), waving one hand (a8), and waving two hands (a9). Representative frames of this dataset are shown in Figure 4(b). The classified results are shown in Table 2.

### 6.3. Evaluation on UCF Sports Action Dataset

The UCF sports action dataset is as follows. This dataset consists of several actions from various sporting events from the broadcast television channels. The actions in this dataset include diving (a1), golf swinging (a2), kicking (a3), lifting (a4), horse-back riding (a5), running (a6), skating (a7), swinging (a8), and walking (a9). Representative frames of this dataset are shown in Figure 4(c). The classified results are shown in Table 3.

### 6.4. Evaluation on Our Action Dataset

Our action dataset is as follows.

We capture the behavior video in the laboratory. It contains five types of human actions (walking (a1), jogging (a2), running (a3), boxing (a4), and handclapping (a5)). Some sample frames are shown in Figure 4(d). The classified results achieved by this approach are shown in Table 4.

### 6.5. Algorithm Comparison

In this case, we compare the proposed method with the three methods: Martínez-Contreras et al. [38], Chaaraoui et al. [39], and Zhang and Gong [40] in four datasets. In Tables 5, 6, and 7, it is obvious that the low recognition accuracy existed in these methods for the complex occlusion situation and the complex beat, motion, and other group actions. The average accuracy in our method is higher than that in the comparative method.

The experimental results show that the proposed approach can get satisfactory results and overcome these problems by comparing the average accuracy with that in [38–40].

## 7. Conclusions and Future Work

In this paper, we present a novel method of human action recognition, which is based on the reconstructed phase space. Firstly, the human body is divided into 15 key points, whose trajectory represents the human body behavior, and the modified particle filter is used to track these key points for self-occlusion. Secondly, we reconstruct the phase space for extracting more useful information from human action trajectories. Finally, we can construct use the semisupervised probability model and Bayes classified method to classify. Experiments were performed on the Weizmann, KTH, UCF sports, and our action dataset to test and evaluate the proposed method. The compare experiment results showed that the proposed method can achieve was more effective than compare methods.

Our future work will deal with adding complex event detection by the phase space-based action representation and action learning and theoretical analysis of their relationship, involving more complex problems, such as dealing with more variable motion and interpersonal occlusions.

## Figures and Tables

**Algorithm 1:**
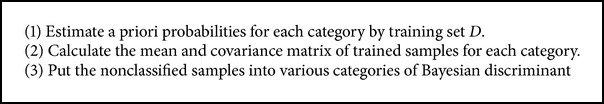


**Table 1:** Confusion matrix for KTH dataset.

	a1	a2	a3	a4	a5
a1	**0.95**	0.01	0.02	0.00	0.01
a2	0.01	**0.93**	0.02	0.10	0.00
a3	0.00	0.02	**0.90**	0.00	0.01
a4	0.01	0.00	0.00	**0.92**	0.30
a5	0.03	0.00	0.02	0.00	**0.82**

**Table 2:** Confusion matrix for the Weizmann dataset.

	a1	a2	a3	a4	a5	a6	a7	a8	a9
a1	**1.00**	0.01	0.02	0.00	0.20	0.00	0.10	0.05	0.02
a2	0.01	**0.96**	0.02	0.03	0.00	0.00	0.00	0.04	0.00
a3	0.00	0.00	**0.80**	0.10	0.13	0.00	0.02	0.01	0.00
a4	0.00	0.01	0.00	**0.95**	0.00	0.20	0.04	0.00	0.00
a5	0.00	0.01	0.00	0.00	**0.85**	0.00	0.00	0.30	0.02
a6	0.01	0.00	0.03	0.00	0.05	**0.91**	0.02	0.00	0.01
a7	0.00	0.03	0.00	0.00	0.01	0.00	**0.94**	0.00	0.02
a8	0.00	0.03	0.04	0.10	0.00	0.00	0.00	**0.98**	0.00
a9	0.00	0.00	0.20	0.00	0.10	0.00	0.00	0.03	**1.00**

**Table 3:** Confusion matrix for the UCF sports dataset.

	a1	a2	a3	a4	a5	a6	a7	a8	a9
a1	**0.97**	0.02	0.01	0.00	0.15	0.00	0.10	0.05	0.02
a2	0.00	**0.95**	0.01	0.00	0.00	0.02	0.00	0.03	0.00
a3	0.01	0.00	**0.82**	0.15	0.10	0.00	0.02	0.02	0.00
a4	0.00	0.00	0.00	**0.92**	0.10	0.10	0.00	0.00	0.00
a5	0.00	0.01	0.20	0.00	**0.88**	0.00	0.00	0.10	0.02
a6	0.01	0.00	0.02	0.00	0.05	**0.93**	0.05	0.01	0.02
a7	0.00	0.04	0.00	0.00	0.00	0.00	**0.92**	0.00	0.02
a8	0.00	0.02	0.03	0.10	0.00	0.00	0.00	**0.97**	0.00
a9	0.00	0.10	0.30	0.04	0.10	0.00	0.00	0.00	**1.00**

**Table 4:** Confusion matrix for our dataset.

	a1	a2	a3	a4	a5
a1	**0.98**	0.00	0.00	0.01	0.02
a2	0.00	**0.96**	0.01	0.00	0.00
a3	0.00	0.02	**0.87**	0.01	0.00
a4	0.00	0.20	0.00	**0.88**	0.02
a5	0.02	0.10	0.00	0.00	**0.86**

**Table 5:** Comparison with other approaches on KTH action dataset.

Method	Average recognition rate (%)
The proposed method	92.30
Martínez-Contreras et al. [38]	89.20
Chaaraoui et al. [39]	91.20
Zhang and Gong [40]	90.60

**Table 6:** Comparison with other approaches on the Weizmann action dataset.

Method	Average recognition rate (%)
The proposed method	89.10
Martínez-Contreras et al. [38]	85.10
Chaaraoui et al. [39]	87.20
Zhang and Gong [40]	85.40

**Table 7:** Comparison with other approaches on UCF sportsaction dataset.

Method	Average recognition rate (%)
The proposed method	91.10
Martínez-Contreras et al. [38]	85.20
Chaaraoui et al. [39]	87.30
Zhang and Gong [40]	88.60

**Table 8:** Comparison with other approaches on our action dataset.

Method	Average recognition rate (%)
The proposed method	90.30
Martínez-Contreras et al. [38]	88.80
Chaaraoui et al. [39]	89.60
Zhang and Gong [40]	87.10
